# Folic acid and zinc improve hyperuricemia by altering the gut microbiota of rats with high-purine diet-induced hyperuricemia

**DOI:** 10.3389/fmicb.2022.907952

**Published:** 2022-07-29

**Authors:** Xuewei Sun, Jie Wen, Baosheng Guan, Jialin Li, Jincheng Luo, Jie Li, Mingyu Wei, Hongbin Qiu

**Affiliations:** ^1^School of Public Health, Jiamusi University, Jiamusi, China; ^2^Heilongjiang Provincial Key Laboratory of Gout Research, Jiamusi, China

**Keywords:** hyperuricemia, uric acid, gut microbiota, folic acid, zinc

## Abstract

A high-purine diet can cause hyperuricemia and destroy the microbial composition of the gut microbiota. Both folic acid and zinc significantly reduce uric acid levels and alleviate hyperuricemia. However, whether the underlying mechanisms are associated with the regulation of the gut microbiota remain unknown. To explore alterations of the gut microbiota related to folic acid and zinc treatment in rats with hyperuricemia in our study. A hyperuricemic rat model was established with a high-purine diet. The effects of folic acid and zinc on uric acid levels were evaluated. Alterations of the gut microbiota related to hyperuricemia and the treatments were evaluated by sequencing using the Illumina MiSeq system. The results demonstrated that uric acid levels dropped observably, and the activities of adenosine deaminase (ADA) and xanthine oxidase (XOD) were downregulated after folic acid or zinc intervention. 16S rRNA gene sequencing-based gut microbiota analysis revealed that folic acid and zinc enhanced the abundance of probiotic bacteria and reduced that of pathogenic bacteria, thus improving intestinal barrier function. PICRUST analysis indicated that folic acid and zinc restored gut microbiota metabolism. These findings indicate that folic acid and zinc ameliorate hyperuricemia by inhibiting uric acid biosynthesis and stimulating uric acid excretion by modulating the gut microbiota. Thus, folic acid and zinc may be new and safe therapeutic agents to improve hyperuricemia.

## Introduction

Hyperuricemia is a chronic metabolic disease related to disordered purine metabolism. In addition, hyperuricemia also is the main factor in occurrence of various diseases, including gout, diabetes, cardiovascular disease, metabolic syndrome, atherosclerosis, Alzheimer’s disease, and chronic kidney disease (Waugh, 2008; Feig, 2014; Kttgen and Kttgen, 2021). Hyperuricemia not only poses a threat to human health but is also becoming a serious public health concern (Paul et al., 2017). Drug treatment currently is the preferred method for clinical management of hyperuricemia, primarily using xanthine oxidase inhibitors and uric acid excretion drugs, including benzbromarone, allopurinol, and febuxostat (Kunishima et al., 2007; Kim et al., 2015; Mehmood et al., 2017). However, these drugs have poor safety profiles and exert side effects, including rash, nausea, vomiting, severe hypersensitivity, and gastrointestinal and renal toxicity (Chen et al., 2020). Therefore, a safer and more effective treatment to lower blood uric acid levels in patients is needed.

Uric acid is the final product of purine metabolism in human bodies (Merriman and Dalbeth, 2011; Liu et al., 2020). Based on the evidence, it is accepted that the kidneys are the main organ for uric acid excretion; they remove about 70% of the total uric acid (Lin et al., 2014; Barsoum and El-Khatib, 2017; Sato et al., 2019). Various studies revealed that 30% of total uric acid is excreted through the gut (Wang et al., 2020, 2021). Blood uric acid is transported into the gut by uric acid transporters in epithelial cells (Matsuo et al., 2014). Subsequently, the uric acid will be excreted from the gut directly, and various gut microbiota will decompose the rest of the uric acid. Growing evidence demonstrates that the gut microbiota and its metabolites occupy a vital position in the pathogenesis of hyperuricemia-related diseases (Rooks et al., 2014; Ananthakrishnan et al., 2017). Therefore, exploring the mechanism of hyperuricemia based on the gut microbiota has become a central research topic.

Folic acid is a water-soluble vitamin B involved in the synthesis of purine, DNA, and hemoglobin. Based on population data, the intake of folic acid has been related to the risk of hyperuricemia (Zhang and Qiu, 2018). Zinc is an essential cofactor for various enzymes and plays important roles in the regulation of inflammatory cytokines and the anti-oxidative stress response (Xu et al., 2015). According to the National Health and Nutrition Examination Survey program, the intake of dietary zinc in American adults (≥20 years of age) correlated with the occurrence of hyperuricemia (Zhang et al., 2018). Xanthine is converted into uric acid, and uric acid is excreted into the gut by the gut microbiota (Sorensen and Levinson, 1975). Folic acid and zinc are some of the vitamins and trace elements that the human body needs to supplement daily, and they will not cause harm to the human body within the range of standard doses. Thus, researching the alterations induced in the gut microbiota by vitamin or trace element therapy may provide candidate drug targets for further in-depth research.

This study aimed to explore the alterations of the gut microbiota induced by hyperuricemia treatment employing folic acid and zinc. 16S rRNA gene sequencing using the Illumina MiSeq platform was employed to study the community structure of the gut microbiota in hyperuricemia rats. Further, we aimed to determine the mechanisms of folic acid and zinc in reducing uric acid levels in hyperuricemia rats.

## Materials and methods

### Hyperuricemia model establishment and drug therapy

A total of forty Sprague–Dawley male rats (2-month-old) weighing 200 ± 20 g were ordered by the Changchun Yisi Laboratory Animal Technology Co., Ltd. (Jilin, China; certification No. SCXK (Ji)-2018-0007). The rats were separated into four groups randomly (10 rats per group): a blank control group (C), a hyperuricemia model group (M), a folic acid treatment group (Y), and a zinc treatment group (Z). The rats were housed individually, with free access to food and water. After 7 days of adaptive feeding (AIN-93M feed provided by Nantong Trophy Co., Ltd., China), three groups were given a hyperuricemia-inducing diet consisting of a mixture of AIN-93M feed and yeast at a 4:1 ratio. A normal diet was provided to the blank control group throughout the experiment.

After establishing the hyperuricemia model successfully, the rats in the folic acid treatment group and the zinc treatment group were intragastrically administered 84 μg/kg folic acid and 4 mg/kg zinc per day for 8 weeks, respectively. Distilled water was given to the rats in the control and model groups. The weights of the rats were measured weekly to calculate the drug dosages.

### Sample collection and storage

After 12 h of the last drug administration, blood samples and stool samples of rats were collected. The collected blood from the hepatic portal vein was centrifuged. The serum samples were frozen at −80°C for serum biochemical index detection, including uric acid, adenosine deaminase (ADA), and xanthine oxidase (XOD) levels. An automated biochemical analyzer (AU5800, Beckman, United States) was used to detect uric acid levels. ADA and XOD were detected using ADA and XOD assay kits (Jiancheng, China) according to the manual’s instructions. Fresh stool samples were collected and stored at −80°C for gut microbiota DNA extraction.

### 16S rRNA gene amplification and sequencing

Genomic DNA of the stool samples was extracted utilizing the Stool DNA Extraction Kit (Qiagen, Germany), and the extracted DNA was purified employing a DNA gel purification kit (Qiagen, Germany). The quality of the extracted DNA was assessed by 1.0% agarose gel and stored at −80°C for sequencing.

The specific primers 338F and 806R were used to amplify the hypervariable region V3–V4 of the 16S rRNA gene (Meng et al., 2020). PCRs were run in 20.0-μl reaction mixtures containing 4 μl 5 × FastPfu Buffer, 2 μl 2.5 mM dNTPs, 0.8 μl forward primer (5 μM), 0.8 μl reverse primer (5 μM), 0.4 μl FastPfu Polymerase, 0.2 μl BSA, 10 μl template DNA, and 1.8 μl ddH_2_O, in an ABI GeneAmp 9700 instrument (ABI, United States). The thermal cycling program was as follows: 95°C for 3 min, 30 cycles of 95°C for 30 s, 55°C for 30 s, 72°C for 45 s, and finally, 72°C for 10 min. The total purified PCR products with a concentration >10 ng/μl and an OD260/OD280 ≈ 1.8 were subjected to sequencing on an Illumina MiSeq platform at Shanghai Meiji Biomedical Technology (Shanghai, China). The sequence data were submitted to the NCBI Sequence Read Archive with accession PRJNA721594.^1^

### Bioinformatical and statistical analysis

After sequencing, Flash software 1.2.11 (Yu et al., 2018) was used to splice pair-end sequences, and the generated data of the gut microbiota were analyzed using the Quantitative Insights Into Microbial Ecology software, v1.9.1 (Caporaso et al., 2010). Clean reads were obtained from the raw reads under the following criteria: reads were truncated at any site receiving an average quality score of <20 bp over a 50-bp sliding window, truncated reads shorter than 50 bp were discarded and the reads containing N base were removed; based on overlap relationship of pair-end reads, pairs of reads were merged into a sequence; barcode of the sequences and primer sequences were used to distinguish samples and obtained effective sequences. The sequencing reads were clustered into operational taxonomic units (OTUs) using UCLUST (Edgar, 2010). The SILVA rRNA database 138 (Quast et al., 2013) was used to perform the taxonomic assignment. The Vegan package (Cheng and Ray, 2014) in R (R Development Core Team, 2015) was used to calculate the alpha-diversity index. Calculated beta-diversity metrics (Bray Curtis) were compared using the ANOSIM measure. The Vegan package in R was used to conduct principal coordinates analysis (PCoA) and non-metric multidimensional scaling based on the beta-diversity. The specific characterization of the gut microbiota was analyzed using the linear discriminant analysis (LDA) effect size (LEfSe) analysis (Segata et al., 2011). LEfSe used a non-parametric factorial Kruskal–Wallis sum-rank test to determine the features with significantly different abundances among groups and used LDA to assess the effect size of each feature, and the threshold on the logarithmic score of LDA analysis was set to 2.0. To compare the key phylotypes of the gut microbiota, Welch’s *t*-tests were used for two-group comparisons in STAMP software 8.30 (Parks et al., 2014). The significant difference between the two groups was obtained after filtering with *p* < 0.05. The PICRUST2 software 2.2.0 (Douglas et al., 2020) was used to infer the metabolic functions of the gut microbiota. To reveal the different predictive functions, the Kruskal–Wallis H test was used for multiple groups comparisons in STAMP software 8.30, *p*-values were adjusted using the Benjamini–Hochberg method to control the false discovery rate (FDR), and an adjusted *p*-value of 0.05 was used as a statistically significant cutoff. The BugBase software (Tonya et al., 2017) was used to predict microbial phenotypes.

All statistical analyses were performed using SPSS 23.0 (IBM, Armonk, NY, United States) and all graphics were made in Prism 7.0 (GraphPad Software, La Jolla, CA, United States). Spearman correlation was used to analyze correlations between biochemical parameters and the major microbial communities; *p* < 0.05 was considered statistically significant.

## Results

### Effects of folic acid and zinc on uric acid levels in rats

The rats remained in healthy during the experimental period, and weekly recording of the body weight and feed consumption of the rats was conducted. Changes in body weight were similar in all groups, without significant differences during the intervention. Rats receiving folic acid and zinc compared with those in the model group had no visible tendency toward drop feed consumption.

**FIGURE 1:**
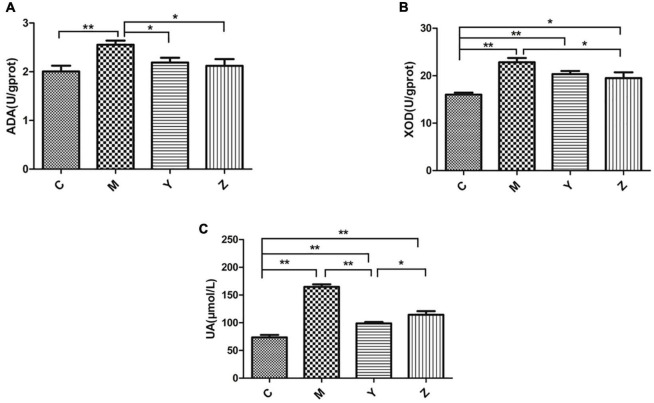
Effects of folic acid and zinc on hyperuricemia-related indicators in hyperuricemia rats. **(A)** The activity of adenosine deaminase (ADA). **(B)** The activity of xanthine oxidase (XOD). **(C)** The level of uric acid (UA). *p < 0.05; **p < 0.01.

The most obvious feature of hyperuricemia is a high uric acid level. The uric acid levels in each group are shown in Figure 1. The uric acid level had a significant increase in the model group compared to the control group (*p* < 0.01), verifying that the hyperuricemia model was established successfully. Interestingly, there was a significant reduction in uric acid levels by the folic acid and zinc treatments, but remained above the normal level (*p* < 0.01). Compared to the model group, the XOD and ADA levels were significantly reduced by the folic acid and zinc treatments (*p* < 0.05), but were also still above the normal levels (Figure 1). Together, these results showed that folic acid and zinc significantly reduce serum uric acid concentrations.

### Treatment effects on gut microbiota diversity of rats

**FIGURE 2:**
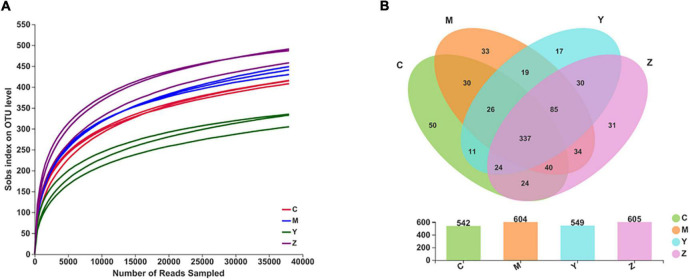
The rarefaction curves and the Venn diagram for each sample in hyperuricemia rats. Panel **(A)** represents the rarefaction curves. Panel **(B)** represents the Venn diagram.

Based on the uric acid results, we selected the rats treated with high doses of folic acid and zinc showing the best pharmacodynamic effects as representatives to carry out the gut microbiota analysis. We used the Illumina MiSeq technology to analyze differences in the gut microbiota composition among the groups. In total, 875,085 sequence reads were detected. With increasing numbers of sequences in the samples, the rarefaction curve gradually flattened, explaining that the sequencing results were abundant to represent the diversity of the samples (Figure 2A). A Venn diagram exhibiting the numbers of OTUs in common between groups and unique to each group is presented in Figure 2B.

**FIGURE 3:**
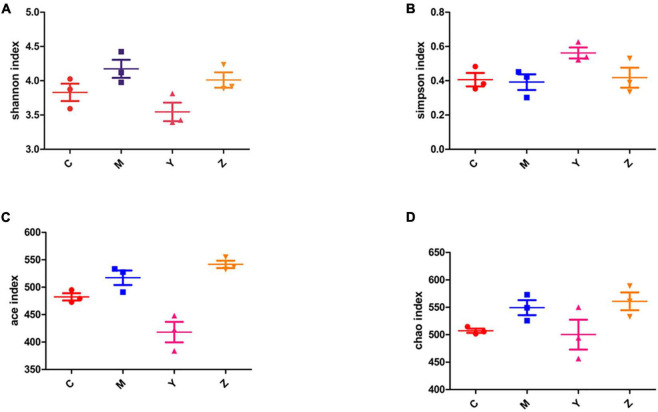
The alpha-diversity index of the gut microbiota in hyperuricemia rats. The alpha-diversity of the gut microbiota based on **(A)** Shannon index. **(B)** Simpson index. **(C)** Ace index. **(D)** Chao index.

Alpha-diversity analysis was used to reveal the microbiota community diversity in each sample. We employed the Chao, Ace, Shannon, and Simpson indices to evaluate alpha-diversity. The Chao and Ace indices reflect community richness, whereas the Shannon and Simpson indices reflect community richness and evenness. The alpha-diversity of the gut microbiota community in the rats is presented in Figure 3. The Shannon, Ace, and Chao indices in the model group exceeded in the control group. The Ace and Chao indices peaked in the zinc treatment group. According to the Simpson index, there was a markedly lower diversity in the model group than in the other groups. However, there was no significant difference among the groups. These results indicated a higher microbial richness in the model group.

**FIGURE 4:**
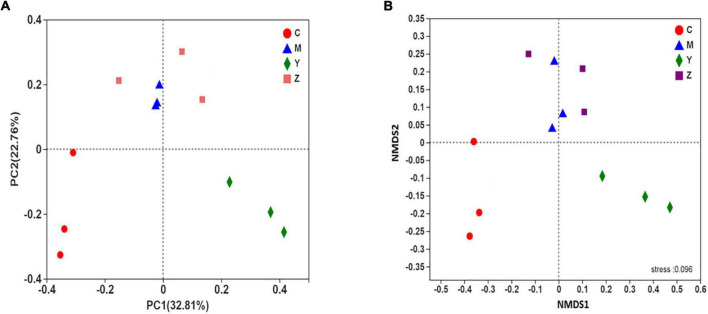
The beta-diversity of the gut microbiota in hyperuricemia rats. The beta-diversity of the gut microbiota based on **(A)** Principal coordinates analysis (PCoA) analysis. **(B)** Non-metric multidimensional scaling plot (NMDS) analysis.

The beta-diversity of the gut microbiota in the rats was explained using principal coordinates analysis (PCoA) and non-metric multidimensional scaling plot (NMDS plot). In addition, the beta-diversity was analyzed using the unweighted UniFrac distance. Based on the PCoA and NMDS, a visible separation was found among the four groups (the control group was located on the left, the folic acid group was located to the right, and the model and zinc groups were located near the middle of the plot), indicating differences in the community composition among the groups (*p* < 0.01) (Figure 4). The microbiota community structure in the folic acid treatment group was clearly different from that in the zinc treatment group, whereas, in the model and zinc treatment groups, the microbiota community structures were closer. These results indicated that folic acid and zinc affected the community composition of the gut microbiota.

### Treatment effects on the taxonomic composition of the gut microbiota

**FIGURE 5:**
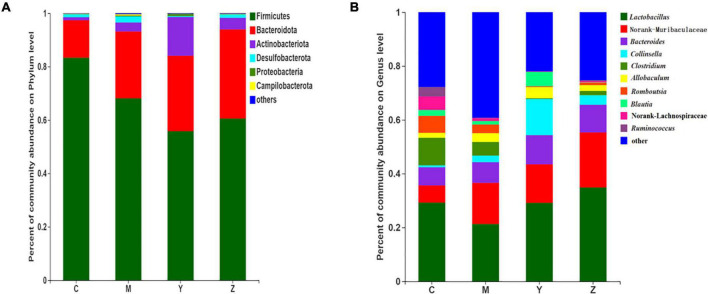
The taxonomic compositions of the gut microbiota in hyperuricemia rats. Relative abundance of the gut microbial community in each group at **(A)** The phylum level. **(B)** The genus level.

To identify specific taxa in the folic acid and zinc treatment groups, relative bacterial abundances were assessed at the phylum and genus levels. The dominant phyla were Bacteroidetes and Firmicutes in each group (Figure 5A). The model group showed remarkable shifts in the gut microbiota composition and structure when compared to the control group, with significant increases in Bacteroides and Actinobacteria and a reduction in Firmicutes. Surprisingly, the gut microbiota (Bacteroides and Firmicutes) of the rats were dominant in the folic acid and zinc treatment groups, but the number of Actinobacteria was sharply increased by nearly three times in the folic acid treatment group. In general, these results verified that folic acid has a major effect on Actinobacteria, but limited effects on Firmicutes and Bacteroidetes.

At the genus level, 10 genera were identified (Figure 5B). *Lactobacillus*, Norank-f-Muribaculaceae, and *Bacteroides* were the most abundant gut microbiota in all groups. The most abundant genera in the control group included *Lactobacillus*, Norank-f-Muribaculaceae, *Bacteroides*, and *Clostridium*. Compared with the control group, Norank-f-Muribaculaceae was notably elevated in abundance, whereas *Collinsella*, *Clostridium*, *Romboutsia*, Norank-f-Lachnospiraceae, and *Ruminococcus* were decreased in abundance in the model group. The microbiota community structures in the folic acid and zinc treatment groups differed from that in the model group. Compared to the model group, *Lactobacillus*, *Bacteroides*, *Collinsella*, and *Blautia* were more abundant, whereas *Clostridium*, *Romboutsia*, Norank-f-Lachnospiraceae, and *Ruminococcus* were little abundant in the folic acid treatment group. Conversely, *Lactobacillus*, Norank-f-Muribaculaceae, and *Bacteroides were* more abundant and *Clostridium, Romboutsia* lower, *Blautia*, and Norank-f-Lachnospiraceae were more abundant in the zinc treatment group. These results indicated the beneficial influences of folic acid and zinc on the abundances of several genera that were influenced by hyperuricemia.

### Treatment effects on key phylotypes in the gut microbiota of rats

**FIGURE 6:**
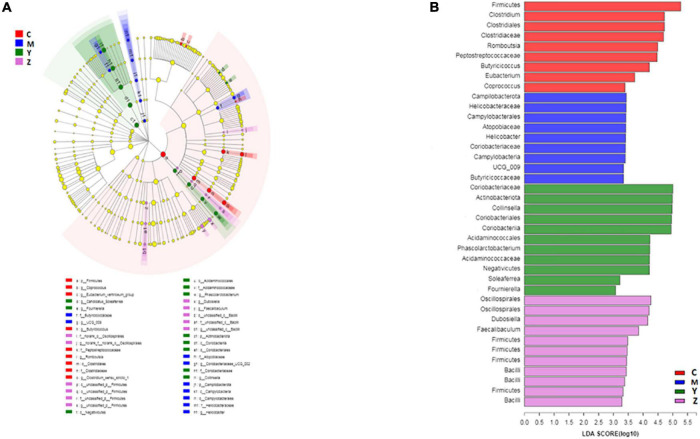
The key phylotypes of the gut microbiota in hyperuricemia rats. LEfSe analysis was used to generate **(A)** Taxonomic cladogram and **(B)** Histogram of linear discriminant analysis (LDA) scores.

**FIGURE 7:**
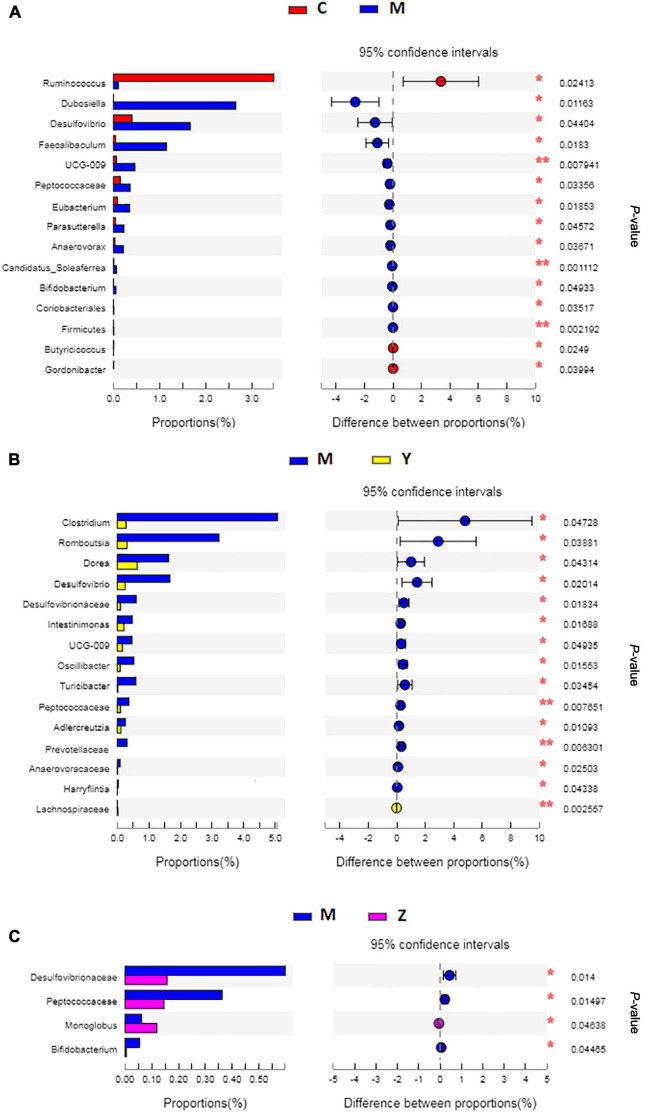
The key phylotypes comparisons in hyperuricemia rats. **(A)** The comparison of the key phylotypes between the control group and the model group at genus level. **(B)** The comparison of the key phylotypes between the control group and the folic acid group at genus level. **(C)** The comparison of the key phylotypes between the control group and the zinc group at genus level. *p < 0.05; **p < 0.01.

To explore the variations of characteristic bacteria in the rats, LEfSe analysis was conducted to detect differences in the abundance of bacterial taxa among the four groups. The taxonomic cladogram and histogram of LDA scores in Figure 6 show the dominant microorganisms in the groups. In the control group, LEfSe revealed that the indicator microorganisms were assigned to *Coprococcus*, *Eubacterium*, *Butyricicoccus*, *Romboutsia*, and *Clostridium* (Figure 6A); *Coriobacteriaceae* and *Helicobacter* were the main microorganisms in the model group; Candidatus *Soleaferrea*, *Fournierella*, *Phascolarctobacterium*, and *Collinsella*, *Dubosiella*, and *Faecalibaculum* were the dominant microbes in the folic acid group and zinc treatment group, separately. As shown in Figure 7, 15 key phylotypes were enriched in the model group versus the control group. The 15 key phylotype species in the folic acid treatment group were significantly different and with distinct characteristics compared with the model group; the most significantly different key phylotypes were *Peptococcaceae*, *Prevotellaceae*, and Norank-f-*Lachnospiraceae*. In the zinc group, four key phylotype species, including *Desulfovibrionaceae*, *Peptococcaceae*, *Monoglobus*, and *Bifidobacterium*, were significantly different and with distinct characteristics as compared with those in the model group.

### Correlations between major operational taxonomic units and biochemical parameters

**FIGURE 8:**
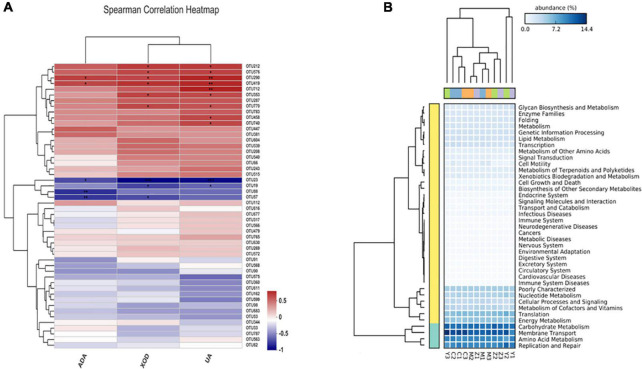
Spearman correlation heatmap and the potential metabolic function of the gut microorganisms in hyperuricemia rats. **(A)** Spearman correlation heatmap revealed the relationship between hyperuricemia-related indicators and the main microbial community. *p < 0.05; **p < 0.01. **(B)** The difference of predicted microbial functions in KEGG pathways among groups.

Spearman correlation was used to reveal the relationships among biochemical parameters and the major microbial communities (OTUs). As shown in Figure 8A, ADA, XOD, and uric acid levels were significantly associated with the bacterial community composition; OTU290 (*Faecalibaculum*) and OTU419 (*Coriobacteriaceae*) were positively correlated with ADA; OTU23 (*Lactobacillus*), OTU88 (*Bacteroides*), and OTU57 (*Anaerostipes*) were significantly negatively correlated with ADA. XOD was significantly positively correlated with variations in OTU290, OTU419, OTU212 (Norank-f-Muribaculaceae), OTU576 (*Allobaculum*), OTU353 (*Erysipelatoclostridium*), and OTU770 (Norank-f-Muribaculaceae), were negatively, albeit not significantly, correlated with the abundances of OTU19 (*Ruminococcus*) and OTU57, and were significantly negatively related to OTU23. OTU290, OTU419, and OTU712 (Norank-f-Muribaculaceae) were significantly positively correlated with uric acid; OTU212, OTU576, OTU353, OTU770, OTU458 (*Dubosiella*), and OTU740 (*Bacteroides*) were positively related to uric acid; OTU23 and OTU19 (*Ruminococcus*) also showed a significantly negatively correlation with uric acid. Interestingly, OTU88 was only significantly correlated with ADA, and OTU712, OTU458, and OTU740 were only significantly correlated with uric acid.

### Changes in the metabolic functions of the gut microbiota of rats

The potential metabolic functions of the gut bacteria were predicted using the PICRUST2 software in accordance with the kyoto encyclopedia of genes and genomes (KEGG) database. Five KEGG pathways in level 1 differed among the four groups, including pathways involved in metabolism, cellular processes, organismal systems, genetic information processing, and environmental information processing. In general, 40 KEGG pathways in level 2 were determined (Figure 8B), and there were significant differences between the control group and the model group. Additionally, KEGG pathways upregulated in the folic acid treatment group were mainly related to amino acid metabolism, carbohydrate metabolism, membrane transport, energy metabolism, nucleotide metabolism, replication and repair, poorly characterized, and translation. In contrast, these pathways were downregulated in the zinc treatment group.

### Predicted phenotypes in the gut microbiota of rats

**FIGURE 9:**
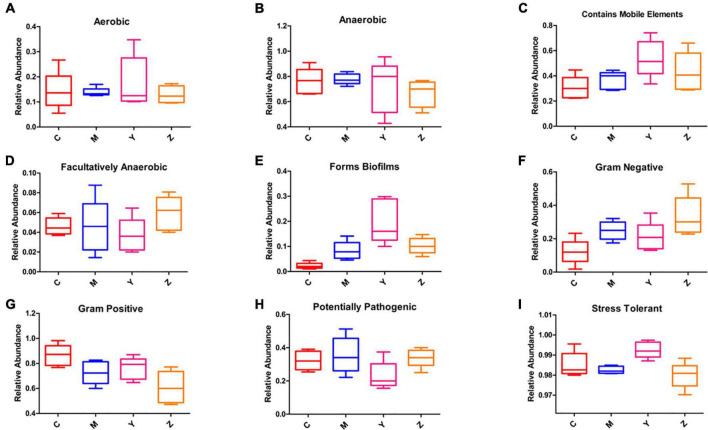
Predicted phenotypes of the gut microbiota in hyperuricemia rats. BugBase was used to predict the proportion of **(A)** Aerobic. **(B)** Anaerobic. **(C)** Contains mobile elements. **(D)** Facultatively anaerobic. **(E)** Forms biofilms. **(F)** Gram-negative. **(G)** Gram-positive. **(H)** Potentially pathogenic. **(I)** Stress-tolerant.

We evaluated the ability of BugBase to predict phenotypes based on the gut microbiota datasets (Figure 9). The majority of predicted phenotypes in the four groups included “aerobic,” “anaerobic,” “contains mobile elements,” “facultatively anaerobic,” “forms biofilms,” “gram-negative,” “gram-positive,” “potentially pathogenic,” and “stress-tolerant,” whereas BugBase predicted the gut microbiota in the four groups to have higher relative abundances of “anaerobic,” “contains mobile elements,” “gram-positive,” “potentially pathogenic,” and “stress-tolerant.” Interestingly, a large alteration was found in the folic acid treatment group, which showed peak proportions of “contains mobile elements” and “forms biofilms” and a very low proportion of “potentially pathogenic.”

## Discussion

A standard model of hyperuricemia is lacking, which has hampered hyperuricemia research. We successfully established a rat model of hyperuricemia by feeding rats a high-purine diet, which mimics the dietary pattern in China. A previous study on the effects and mechanisms of hyperuricemia suggested that hyperuricemia therapy may be targeted not only on inhibiting uric acid synthesis but also on promoting uric acid excretion (Wang et al., 2017; Chen et al., 2021). In our study, increased uric acid levels were observed in the model group, whereas the folic acid and zinc treatments suppressed the activity of enzymes (ADA and XOD) involved in purine metabolism to inhibit the synthesis of uric acid, thus effectively lowering uric acid levels.

Studies showed that the gut is vulnerable to hyperuricemia (Gul and Zager, 2018), and 30% of uric acid in humans is excreted through the gut (Agnoletti et al., 2021; Han et al., 2021). Numerous gut microbiota species can secrete the primary enzymes involved in oxidative purine metabolism (Sathisha et al., 2011; Crane et al., 2013); therefore, we inferred that the gut microbiota has an important influence on the onset and development of hyperuricemia. In general, gut microbiota alterations are observed after drug treatments, which may contribute to disease alleviation (Wilson and Nicholson, 2016). Our study results suggested that supplementation of folic acid or zinc can make a significant impact on the treatment of hyperuricemia.

To explore the roles of folic acid and zinc in regulating the gut microbiota in hyperuricemia and in promoting uric acid excretion, we treated hyperuricemia with folic acid or zinc and observed the gut microbiota structure. Folic acid and zinc could restore the alterations in the gut microbiota diversity caused by the high-purine diet. Similar findings have been reported previously (Ivarsson et al., 2014). In our study, significant differences were observed in the alpha-diversity and beta-diversity of the gut microbiota among the four groups, indicating that the anti-hyperuricemic effects of folic acid and zinc are, at least in part, dependent on the gut microbiota. Additionally, various gut microbiota phyla were correlated with ADA, XOD, and uric acid levels, corroborating that uric acid is also regulated and excreted by the gut microbiota.

Abnormal uric acid excretion in the gut is associated with alterations in the gut barrier, which is essential to maintain the balance between the host and the gut microbiota (Gul and Zager, 2018; Guo et al., 2019; Lv et al., 2020). Studies showed that a healthy gut barrier can availably prevent pathogenic bacteria and harmful substances from entering the intestinal mucosa, thereby maintaining the gut ecological environment (Xia et al., 2017). As probiotic gut bacteria, *Lactobacillus* species can decompose inosine and guanosine to inhibit uric acid biosynthesis, protecting the gut barrier (Bonifacio and Jesús, 2010). Our results showed that the model group evidently dropped relative specie numbers of *Lactobacillus*, whereas folic acid and zinc reversed these alterations. *Romboutsia*, a valuable gut biomarker, made a key contribution to maintaining the gut environment of the healthy host (Spor et al., 2011; Mangifesta et al., 2018). The abundance of *Romboutsia* was decreased in the model group, surprisingly, folic acid or zinc treatment failed to restore *Romboutsia* abundance, indicating that *Romboutsia* cannot survive in a gut environment disturbed by a high-purine diet. We conclude that folic acid and zinc can alleviate hyperuricemia by raising the abundance of probiotic bacteria in the gut and improving gut barrier integrity.

Healthy gut functioning can be promoted by increasing probiotic species that maintain the gut barrier function, and by decreasing the pathogenic bacteria that damage the gut mucosa (Zhou et al., 2021). Our results suggest that the uric acid-lowering effect and the alleviation of gut barrier permeability in hyperuricemia can be attributed to a decrease in pathogenic bacteria in the gut (Meng et al., 2020). Based on the gut microbiota phenotypes predicted in this study, we infer that folic acid and zinc can reduce pathogenic intestinal bacteria, thus maintaining intestinal barrier function. In line herewith, Guo et al. (2016) have reported that an increase in pathogenic gut bacteria may be in charge of high uric acid levels, and hyperuricemia can alleviate by reducing intestinal barrier function damaged by pathogenic bacteria. Hence, the gut microbiota also makes a key contribution to elevating uric acid levels in hyperuricemia.

Two different drug therapies were used to reduce uric acid. Folic acid and zinc exert different influences on the gut microbiota in rats with hyperuricemia, which may depend on their different mechanisms of reducing uric acid levels in hyperuricemia. Consistent with our findings, studies have confirmed that changes in Bacteroidetes and Firmicutes of the gut have significant effects on the metabolism in hyperuricemia (Yu et al., 2018). We found that alterations in Firmicutes and Bacteroidetes induced by the high-purine diet were restored upon intervention with folic acid or zinc (Lima et al., 2015; Peng et al., 2015), influencing lipid, amino acid, nucleotide, carbohydrate, and energy metabolism.

## Conclusion

In summary, folic acid and zinc effectively relieve hyperuricemia, on the one hand, by inhibiting the synthesis of uric acid *via* reducing ADA and XOD activities, and on the other hand, by promoting uric acid excretion by changing the gut microbiota composition. Folic acid is more effective in reducing uric acid levels than zinc. In the future, further study on the gut microbiota correlated with folic acid and zinc treatments may help identify biomarkers of alleviation of hyperuricemia that may be useful in hyperuricemia therapy, further clarifying the molecular mechanisms of folic acid and zinc in regulating the gut microbiota to provide a theoretical foundation for their potential use in the treatment of hyperuricemia.

## Data availability statement

The datasets presented in this study can be found in online repositories. The names of the repository/repositories and accession number(s) can be found in the article.

## Ethics statement

The animal study was reviewed and approved by Ethics Committee of Jiamusi University School of Public Health.

## Author contributions

XS and JL conceived and designed the research. XS, JW, MW, and JLi engaged in experimental operations. HQ and BG analyzed the data of the entire work. XS wrote the manuscript. JCL revised the draft. All authors have checked the final manuscript, contributed to the article, and approved the submitted version.

## Footnotes

1
https://www.ncbi.nlm.nih.gov/bioproject/PRJNA721594
